# Generic amyloidogenicity of mammalian prion proteins from species susceptible and resistant to prions

**DOI:** 10.1038/srep10101

**Published:** 2015-05-11

**Authors:** Sofie Nyström, Per Hammarström

**Affiliations:** 1 IFM-Department of Chemistry, Linköping University, SE-581 83 Linköping, Sweden

## Abstract

Prion diseases are lethal, infectious diseases associated with prion protein (PrP) misfolding. A large number of mammals are susceptible to both sporadic and acquired prion diseases. Although PrP is highly conserved and ubiquitously expressed in all mammals, not all species exhibit prion disease. By employing full length recombinant PrP from five known prion susceptible species (human, cattle, cat, mouse and hamster) and two species considered to be prion resistant (pig and dog) the amyloidogenicity of these PrPs has been delineated. All the mammalian PrPs, even from resistant species, were swiftly converted from the native state to amyloid-like structure when subjected to a native condition conversion assay. The PrPs displayed amyloidotypic tinctorial and ultrastructural hallmarks. Self-seeded conversion of the PrPs displayed significantly decreased lag phases demonstrating that nucleation dependent polymerization is a dominating mechanism in the fibrillation process. Fibrils from Aβ1-40, Aβ1-42, Lysozyme, Insulin and Transthyretin did not accelerate conversion of HuPrP whereas fibrils from HuPrP90-231 and HuPrP121-231 as well as full length PrPs of all PrPs efficiently seeded conversion showing specificity of the assay requiring the C-terminal PrP sequence. Our findings have implications for PrP misfolding and could have ramifications in the context of prion resistant species and silent carriers.

Prion diseases strike more than 50 mammalian species from a vast number of subfamilies^1^, among them man, sheep and cow. Massive evidence supports misfolding and aggregation of the prion protein (PrP) as causative for disease progression irrespective of if the disease is sporadic, inherited or transmitted. Transmission of prion disease between individuals within the same species as well as between different mammalian species has been reported. A notorious mammalian prion disease is bovine spongiform encephalopathy (BSE) in cattle^2^. In rare instances cattle displayed a sporadic form of BSE named atypical BSE or bovine amyloidotic spongiform encephalopathy (BASE)^3^,^4^. Accidental transmission of BSE during the epidemic in the 1980ies resulted in the outbreak of variant Creutzfeldt-Jakob’s disease (vCJD) in humans in the 1990ies^5^,^6^. This in turn raised concern that transmissible spongiform encephalopathies (TSEs) from other domestic species could arise and be transmissible to human or from animal to animal. Cats have been shown to be susceptible to BSE prion infection manifested as Feline spongiform encephalopathy (FSE)^7^. Transmission of BSE to dog and pig, have proven poorly successful despite several experimental and, most likely, undeliberate field attempts. The lack of reports of prion infection in dogs despite the notion that BSE tainted material most certainly has been included in the food supply of domestic dogs can also be interpreted as dogs being resistant to prion infection. Experimental infection of pigs with BSE was poorly successful^8^ and surveying for TSE in pigs fed with meat and bone meal provided no positive results^9^. Recent data suggested that although conversion of brain derived canine PrP (CaPrP) is reluctant to misfold into proteinase K (PK) resistant conformers it is possible to obtain dog derived prion strains that are transmissible to transgenic mice expressing bovine PrP^10^ albeit the question of variation in titers is not fully clear. The putatively prion disease resistant rabbit was also recently shown to possess prion replicating capability, further questioning the concept of prion resistant mammals^11^.

The apparent species barrier has in part been attributed to the primary structure of both inoculum and host. Comparison of the amino acid sequence and native three dimensional fold of prion proteins derived from different mammalian species revealed that there are small differences between these^12^,^13^,^14^,^15^,^16^,^17^,^18^. Misfolded PrP aggregates generated *in vivo* were categorized into different strains based on their biochemical properties as well as the phenotypic expression of disease (reviewed in^19^). One primary structure could give rise to several different strains and the strain specific features could be transmitted from donor to host regardless of primary structure. Inoculation of genetically identical experimental animals using inoculum derived from the same sequence but manifesting as different strains suggested that the species barrier is more dependent on prion strain than on the amino acid sequence of the host and donor. Transgenic mice expressing human PrP (HuPrP) were highly susceptible to inoculation with sporadic or iatrogenic CJD whereas wild type mice were not^20^. On the contrary, the wild type mice were susceptible to variant CJD, which had significantly lower impact on the transgenic animals expressing HuPrP^20^. The same type of strain specific species barriers were shown for BSE and atypical BSE strains transmitted to hamster^21^ cynomoglus macaque^22^ and wild type mice^23^.

Recent studies have shown that recombinant human prion protein in its native conformation but not recombinant prion proteins from other species could inhibit formation of PK resistant PrP^Sc^ in Protein Misfolding Cyclic Amplification (PMCA) using human substrate^24^, demonstrating that the glycosylation pattern in combination with the sequence might modulate prion formation. Notwithstanding, the development of protocols for fibrillation of the prion protein under near native conditions^25^,^26^ has enabled seeding of various PrP sequences with different seeds without generating results biased for fibril stability and denaturant induced conformational rearrangements. In this reductionist study we used full-length recombinant mammalian PrPs (PrP) with the unstructured domain intact. PrPs from human (HuPrP), bovine (BoPrP), porcine (PoPrP), feline (FePrP), canine (CaPrP), murine (MoPrP), hamster (HaPrP) were used to investigate fibril formation.

## Results

### PrPs spontaneously form amyloid-like aggregates

**Figure 1:**
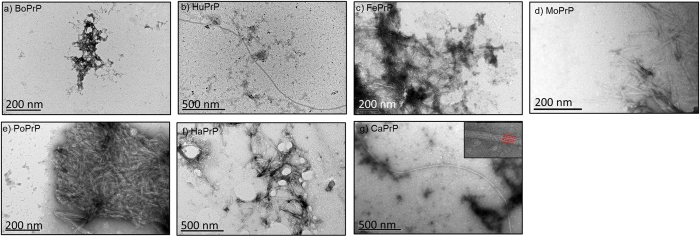
Ultrastructure of PrP aggregates by negative stain transmission electron microscopy. (**a**-**g**)Samples from spontaneous fibrillation reactions collected after 24 hours (BoPrP 48 hours). Aggregates with fibrous morphology were present in all samples except for **a**) BoPrP which displayed morphologically disordered aggregates but no defined amyloid filaments.

**Figure 2:**
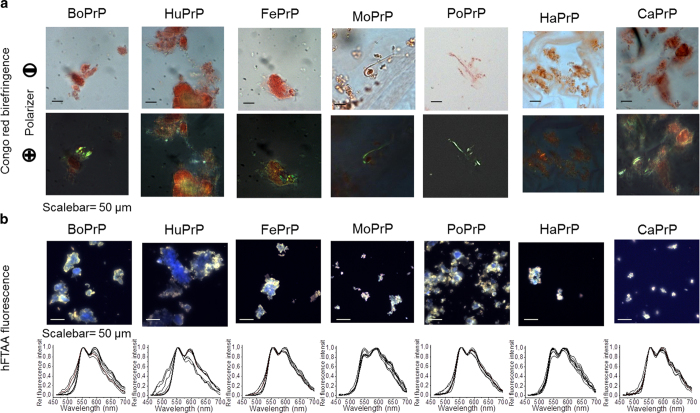
Microscopy of mammalian PrP aggregates stained with amyloid dyes. (**a**) All PrP sequences were stained with Congo red and displayed anomalous colors i.e. birefringence under crossed polarizers. (**b**) All PrP sequences display amyloidotypic hFTAA fluorescence spectra (peaks at 545 nm and 585 nm indicate amyloid structure) when analyzed using hyperspectral imaging^32^. The spectral profile of BoPrP and HuPrP display higher spectral heterogeneity indicating more variable fibril morphology for these sequences compared to the other PrPs^34^.

The proteins in the study were subjected to a native condition conversion assay (NCCA)^25^,^26^. Here the natively folded protein in buffer with physiologically relevant pH and salt concentrations in the absence of denaturing agents or detergents are subjected to intense agitation at 37 °C. Transmission electron microscopy (TEM) was performed to examine the ultrastructural properties of the PrP aggregates that had formed at the endpoint (24 h) of unseeded fibrillation (Fig. 1a–g) as well as during the seeded reaction (Fig. S1). PrP aggregates displayed both ordered micrometer long unbranched fibrils and the presence of dense morphologically disordered aggregates, as shown for HuPrP and CaPrP (Fig. 1b,g). The long mature striated fibrils of CaPrP with a diameter of 15–20 nm were composed of 4 apparent individual parallel protofilaments each with a diameter of 4.5-5 nm (Fig. 1g inset). Aggregates composed of co-assembled shorter fibrils and disordered aggregates were also observed (Fig. 1g). Clustered shorter fibrous aggregates with a high degree of lateral assembly with protofilaments with a diameter of 4-5 nm were evident for FePrP, MoPrP, PoPrP and HaPrP (Fig. 1c–f). Predominantly, morphologically disordered aggregates were observed for BoPrP (Fig. 1a). Overall the ultrastructural properties of the in vitro formed fibrils are compatible with reports on mouse derived fibrils from both *in vitro* and *in vivo* preparations, where single, double and quadruple protofilaments with 4-6 nm diameters^27^ have been observed and widths up to 25 nm have been reported^28^,^29^. Ultrastructural polymorphism has been observed in most cases^27^,^28^,^29^,^30^. Fibrils from the endpoint of each spontaneous fibrillation reaction were stained with Congo red (CR) and analyzed with bright field microscopy using crossed and open polarizers to assess amyloid content in the samples. Samples from all PrP sequences displayed Congo red birefringence, indicating that amyloid-like fibrils of recombinant prion proteins from all the included mammalian species had formed using the conversion assay (Fig. 2a). For further verification, the PrP aggregates were stained with the amyloid specific fluorescent probes thioflavin T (ThT) and hepta-formyl thiophene acetic acid (hFTAA). All PrP aggregates were hFTAA positive with the tinctorial amyloid hallmark of double peak at 545 nm and 585 nm in the emission spectrum (Fig. 2b)^31^,^32^. The hFTAA probe has previously been shown to be useful in distinguishing PrP deposits within prion infected mice^33^. Some heterogeneity of the hFTAA emission spectra were apparent, indicating structural differences within HuPrP and BoPrP whereas PoPrP, CaPrP, FePrP, MoPrP and HaPrP appeared more homogenous although the spectral range is broad^34^. The results from hFTAA fluorescence while at low resolution implicates that there could be polymorphic sites for the probe to bind in BoPrP and HuPrP but most importantly that all PrPs have a substantial fraction with amyloid-like structure. The results from the amyloid-like structural analyses of fibrillated PrPs are summarized in Table 1.

### All purified PrPs are natively folded prior to fibril formation

**Figure 3:**
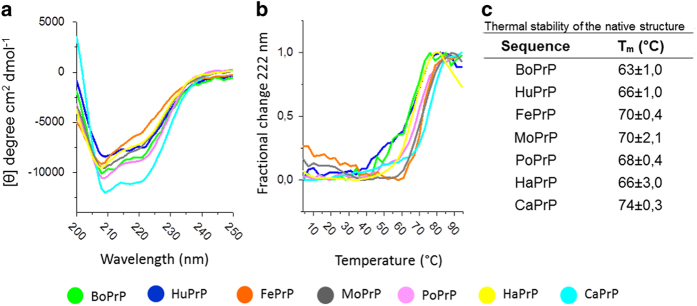
Circular dichroism spectroscopy of mammalian PrPs. (**a**) Far UV CD spectra at 4 °C reveal high α-helical content and indicate natively folded PrPs. (**b**) Thermal denaturation monitored at 222 nm shows cooperative unfolding and some difference in thermal stability between the PrPs. (**c**) Tm values calculated according to^60^. Data was recorded from samples in a 1 mm cuvette with 5 μM PrP in 100 mM sodium phosphate, 50 mM NaCl, 50 mM KCl, pH 7.4.

Following PrP preparation from inclusion bodies, on-column refolding, and monomer isolation by gel filtration^25^, the proteins were studied by circular dichroism (CD) spectroscopy to verify natively folded PrP. All PrPs are rich in α-helical structure as displayed by CD spectra displaying negative peaks at 208 and 222 nm (Fig. 3a). Structural homology beween PrPs of diverse species are expected from NMR studies done by Wüthrich and co-workers^13^,^14^,^15^,^16^ (more below). Structural comparisons of the different PrPs deduced from our CD spectra in the spectral range 200-250 nm was deemed insufficient to draw conclusions thereof. Temperature denaturations showed cooperative unfolding transitions of all PrPs and indicate that the proteins are homogenous and well folded with fully formed disulfide bonds prior to thermal denaturation. Albeit thermal denaturation should preferentially be a fully reversible process to allow for accurate thermodynamic analysis, the midpoint of thermal denaturation (T_m_) is a useful measure of thermal stability. The thermal stability was rather high with T_m_s varying between 63 °C (for BoPrP) to 74 °C (for CaPrP) (Fig. 3b,c).

### Fibril formation kinetics

**Figure 4:**
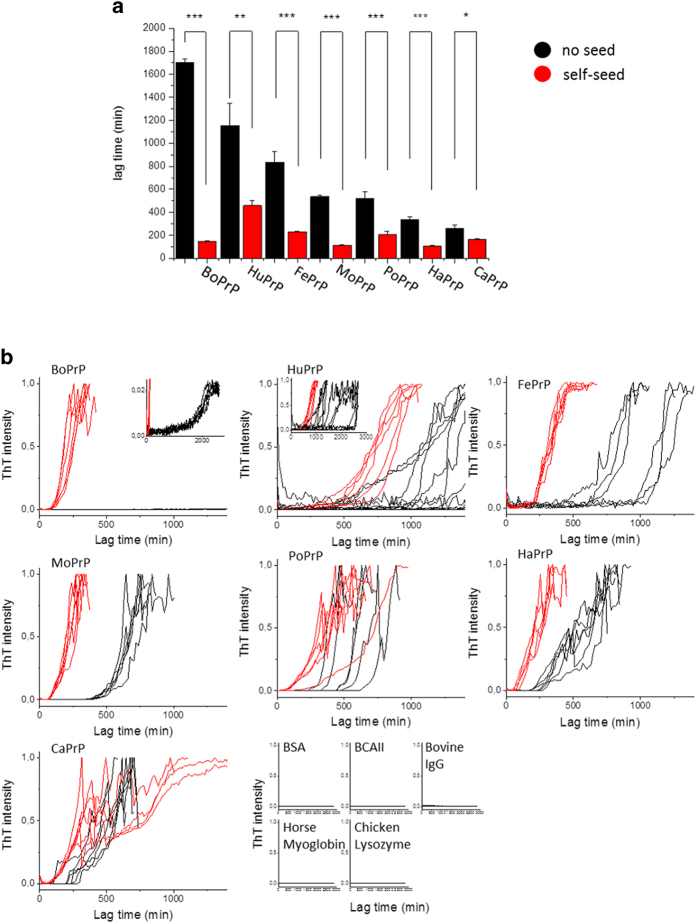
Fibrillation kinetics monitored by ThT fluorescence. **a**) Summary of lag times for unseeded (black) and seeded (red) conversion for PrPs (significance as calculated by paired *t*-test. Significance denoted with stars where * indicates p < 0.05, ** indicates p < 0.005 and *** indicates p < 0.001) **b**) Normalized kinetic traces for unseeded (black) and seeded (with 1% preformed fibrils) (red) fibril conversion of all PrPs. The intensities have been normalized to the maximum value of each trace except for BoPrP which was normalized to the average maximum value for all the other PrPs. One out of 21 BoPrP samples converted similar to the other PrPs and has been retracted from the plots. Unseeded HuPrP samples from several experiments have been pooled. **c**) Kinetic traces for the globular control proteins as noted in the figure, intensities normalized to the average maximum values of the PrPs (excluding BoPrP).

**Figure 5:**
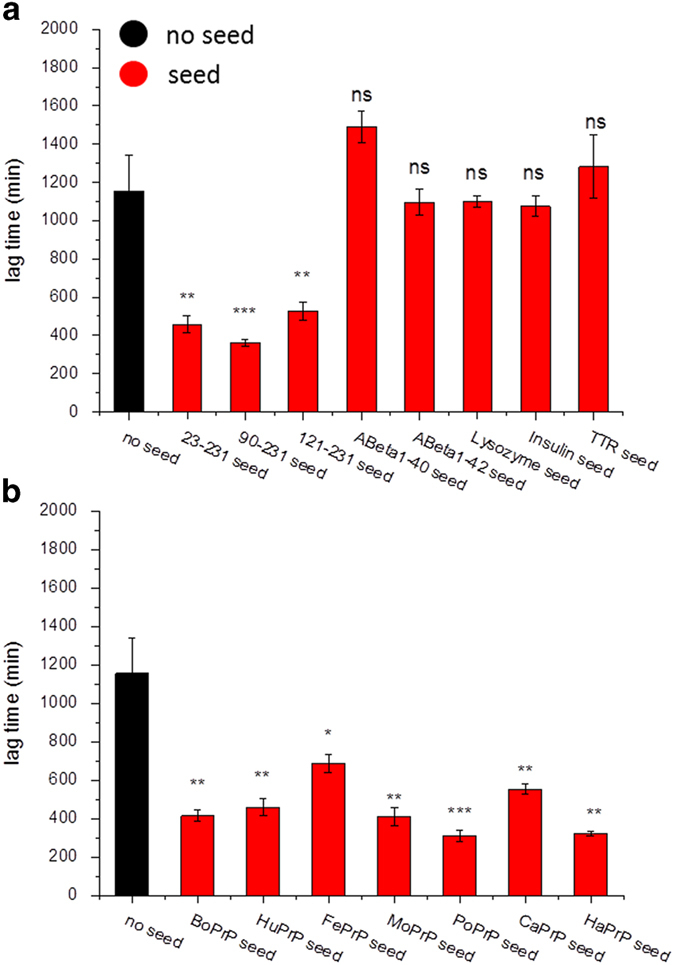
Seeding specificity of HuPrP fibril formation as effects on lag time. **a**) Full length HuPrP can be seeded with 1% preformed fibrils of HuPrP 23-231, 90-231 and 121-231. It cannot be seeded by fibrils from Aβ 1-40, Aβ 1-42, Lysozyme, Insulin or Transthyretin (TTR). **b**). HuPrP can be seeded with 1% preformed fibrils of all other mammalian PrPs of the study.

NCCA performed while monitored by ThT fluorescence *in situ* allows for observation of the fibrillation kinetics^25^. The kinetic trajectories were characterized by lag, growth and equilibrium phases. The median lag times for spontaneous conversion of the PrP into amyloid-like fibrils varied between ~3 to ~26 hours (Fig. 4a,b). CaPrP and PoPrP sequences displayed among the fastest spontaneous conversion rate whereas BoPrP and HuPrP were the slowest. BoPrP displayed very low ThT intensity compared to fibrils generated from the other sequences, however the sigmoidal trajectory (Fig. 4b, inset) is indicative of amyloid fibril conversion. None of the control proteins (Fig. 4b) converted within 48 hours under identical conditions. We have previously shown that fibrillation of HuPrP is a nucleation dependent polymerization process, where efficient self-seeding with preformed fibrils dramatically shortens the lag time and reduces the variation of lag times compared to the unseeded reactions^25^. Here seeding of PrP was applied by adding 1% seed from the end-stage of unseeded reactions before starting new fibrillation reactions. The lag times were significantly reduced for all PrPs (Fig. 4a,b). BoPrP showed the largest decrease in lag time and a dramatic increase in ThT intensity upon seeding, demonstrating its sensitivity to seeded conversion *in vitro*. TEM micrographs of samples from seeded conversion of all PrPs revealed that similar structures, both dense aggregates and protruding fibrils, were present in all the reactions regardless of primary structure (Fig S1). To test if the seeding assay was specific and to probe for structural elements within the PrP sequence important for seeding efficiency we seeded in addition to full length HuPrP with fibrils formed from HuPrP 90-231 and HuPrP 121-231 as well as with fibrils from five other amyloid proteins. The three fibrils derived from HuPrP alloforms showed efficient seeding, whereas none of the heterologous amyloid fibrils were efficient seeds (Fig. 5a). Interestingly seeding of HuPrP with fibrils formed from the other mammalian PrPs was as efficient as for self-seeding (Fig. 5b) suggesting promiscuous seeding of in vitro PrP amyloid-like fibrils. This result could appear somewhat controversial but it is well established that recombinant HaPrP can be seeded with a variety of prions from deer, mice and humans to form fibrils *in vitro* in assays similar to ours^35^.

## Discussion

Misfolding of PrP is a key determinant for prion disease regardless of the disease being sporadic, inherited or acquired. While recombinant PrP amyloid fibrils such as those described herein have low infectivity titers compared to prions formed *in vivo* there are studies showing infectivity of such preparations in hamsters and transgenic mice^36^,^37^,^38^,^39^,^40^,^41^,^42^,^43^,^44^. To understand the requirements for PrP misfolding in detail, it is important to understand the basic behavior of this crucial protein for prion disease. The scope of this study was to investigate if the capacity of PrP replication as amyloid fibrils is sequence dependent. Hereby a possible explanation for lack of prion disease in pigs, dogs and putatively silent carriers^11^ being due to inability of the respective PrP sequence to misfold into amyloid-like structure was addressed. In this study, seven PrPs were prepared as well folded proteins with rather high thermodynamic stability that all displayed a cooperative thermal denaturation, indicating well folded, homogenous proteins. The PrP sequences included in the study were all highly amyloidogenic and were readily either by spontaneous or by seeded conversion misfolded into amyloid-like Congo red, hFTAA and ThT positive fibrils with ultrastructural fibrous morphology. BoPrP diplayed a markedly slower spontaneous conversion rate, resulting in low ThT fluorescence intensity when compared to the other six PrP sequences. Midpoints of thermal denaturation (T_m_) did not correlate with kinetics of fibril formation.

To scrutinize if fibril formation followed the nucleation-dependent polymerization mechanism, preformed fibrils were added at the start of the reaction in so called seeding assays. Most pronounced effects were seen upon seeding the BoPrP because the spontaneous fibrillations of this sequence showed long lag times and displayed low ThT intensities when compared to the other sequences. Analogous reverse correlation between spontaneous amyloidogenesis and seeding propensity has previously been shown for the artificial mutant M129W in the context of HuPrP_90-231_ when compared to other substitutions in the same position^25^. We attributed this feature to the variety of fibril morphologies, many of which are too non-repetitive to bind ThT, which can coexist in a test tube where the kinetics is slow. These immature prefibrillar assemblies can putatively with ease fragment and rearrange into new seeds that nucleate nascent fibrils and effectuate secondary nucleation^45^.

**Figure 6:**
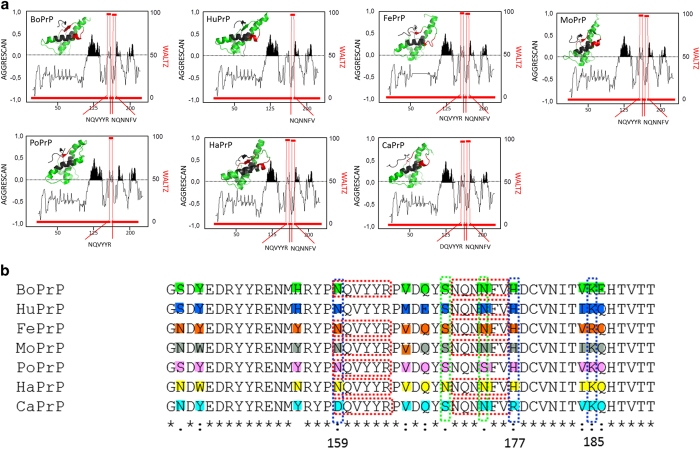
Comparison of mammalian PrP sequences. **a)** Prediction of aggregation prone sequences (AGGRESCAN^46^) and amyloidogenicity (WALTZ^47^) for BoPrP, HuPrP, FePrP, MoPrP, PoPrP, HaPrP, and CaPrP. Continuous stretches with an aggrescan score >1 are regarded as aggregation prone and these peaks are highlighted in black. The PDB structures for each sequence with the predicted amyloidogenic stretches marked in red and aggregation prone sequences in black (PDB entries: BoPrP 1DWY, HuPrP 1QM2, FePrP 1XYJ, MoPrP 1XYX, PoPrP 1XYQ, HaPrP 1B10, and CaPrP 1 XYK^14^,^15^,^16^,^17^,^18^). (**b**) Primary sequence alignments of the polymorphic region implicated as important for disease susceptibility. Stars indicate identical residues colon denotes conserved residues and points mark non-conserved mutations. Mismatched residues are marked in color. The red boxes highlight amyloidogenic stretches according to the WALTZ prediction tool, the blue boxes highlight amino acids modifying disease susceptibility according to^57^ and the green boxes indicate the positions 170 and 174 involved in “rigid loop” formation^55^.

**Figure 7:**
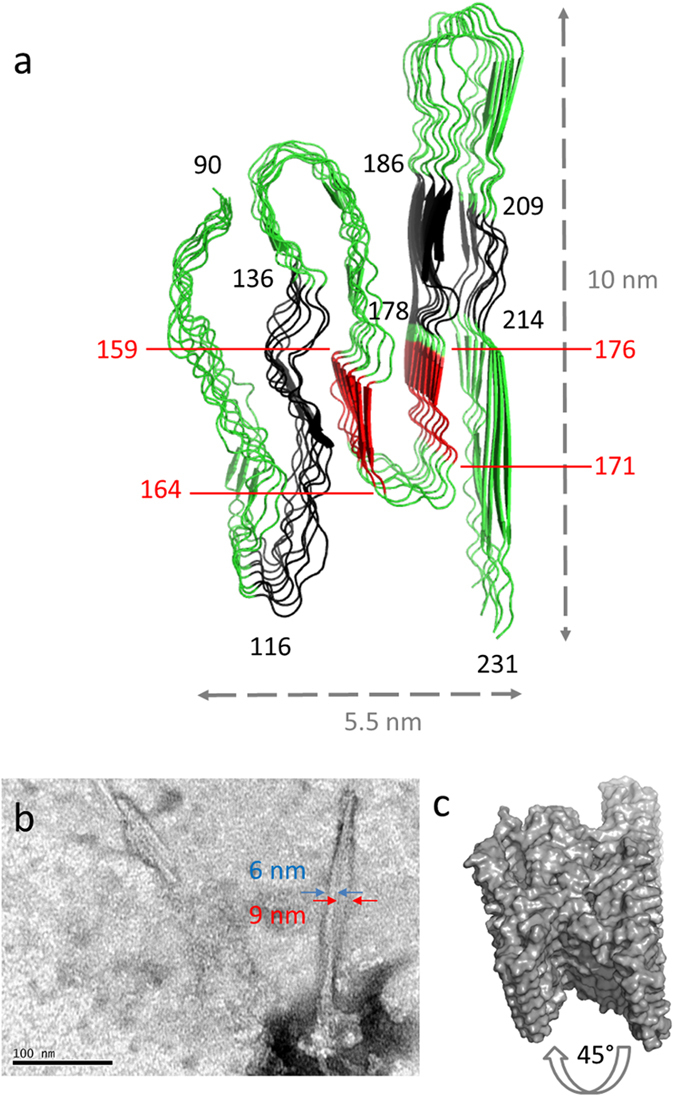
Structural compatibility of diverse mammalian PrPs within amyloid fibrils based on in-register cross β-sheet of PrP 90-231. **a**) Conformational compatibility within the fold of PrP within an amyloid fibril. Black colors denote aggregation prone sequences from AGGRESCAN and red colors denote predicted amyloidogenic sequences by WALTZ (as shown in Fig. 6). The figure was made using PyMol and the pdb coordinates PIRIBS-A-EM-ND.pdb including eight protomer chains from the model proposed by Groveman *et al.* 2014 based on HaPrP90-231. **b**) Negative stain TEM of an individual fibril of HuPrP23-231 from a self-seeded reaction shows a morphotype compatible with the proposed single protein molecule cross section of the model in a similar to that reported by Groveman *et al.* 2014 for brain derived fibrils from MoPrP. **c**) Surface rendering of a tilted fibril model (same as in a) to demonstrate the compatibility of the structural model with the experimental data in b.

The recombinant proteins used for the experiments, lack post-translational modifications and hence show only primary sequence dependence on aggregation and fibrillation behavior without invoking the complexity of partial and diverse glycosylation branching and membrane attachment. The prion protein is well conserved between the mammalian species. Structural studies of the PrP sequences reveal their overall similarity in fold and primary structure (Fig. 6a,b, Fig. S2) and the seven PrP sequences show high homology (80-90%) (Fig. S2). However there are some deviating amino acids at key positions. Differences and similarities between the sequences were compared using AGGRESCAN^46^ and WALTZ^47^ algorithms for identification of putative aggregation prone and amyloidogenic sequence stretches respectively (Fig. 6a,b). The algorithms rendered simplistic information about aggregation and amyloidogenicity of linear stretches of amino acid sequences. Taken together the prediction results indicated similar propensity for aggregation whereas a tentatively different amyloidogenicity of the various sequences. Notwithstanding, the WALTZ predictions must be taken with caution since there are known fibril forming fragments of PrP e.g. HuPrP23-144^48^ which were not noted in the predictions. Several structural models for infectious prions and PrP amyloid fibrils have been proposed but consensus is lacking^49^,^50^. The lack of a defined high resolution misfolded PrP structure is perhaps expected due to the knowledge of prion strains presenting various disease phenotypes^19^. Importantly various strains are also associated with variations in PrP aggregate structure^33^, likely reflecting polymorphs dictating its biological activity. How this relates to the *in vitro* formed amyloid structures is an open question. Notwithstanding, there are commonalities in structural models particularly for *in vitro* formed PrP amyloid fibrils. The majority of models of *in vitro* formed PrP fibrils support a cross-β sheet comprising parallel in-register β-strands of the C-terminal domain^51^, which may stretch all the way from residue 90 to 231^27^, where the C-terminal 166-231 residues are fully buried in the fibril core. Most data support a more flexible N-terminal part (90-125) within the fibril fold. Annealing of the N-terminal sequence involving residues starting from position 80-90 has been shown to occur in *in vivo* formed PrP aggregates^52^. Our seeding data of HuPrP using N-terminally truncated PrPs showed that the 121-231 part of PrP is sufficient to maintain seeding activity (Fig. 5a). Plotting the aggregation prone and amyloidogenic sequences on top of a recently proposed fibril model of HaPrP 90-231^27^ visualizes these discrete sections within the fibril core (Fig. 7a) and how these could be aligned in relation to each other. This model is experimentally compatible with some fibril morphotypes that we observed by TEM for full length HuPrP (Fig. 7b) which are similar to the “selleri stalk” structure described for anchorless MoPrP fibrils formed *in vivo*^30^, suggested to involve a single PrP molecule per repetitive unit within the fibril (Fig. 7b,c). Further support for this model from a structural perspective stems from the high sequence homology between the different mammalian PrPs studied in this report suggesting that many PrP sequences would be compatible with this fold. Accordingly also from our experimental data we observed efficient cross-seeding activity of PrPs from the different sequences which accelerated fibrillation through nucleation dependent polymerization (Fig. 5b). These findings also propose that the species barrier which is well established *in vivo* to cause disease is less stringent for *in vitro* fibrillation. Perhaps amyloidogenicity can be detached from neurotoxicity. There are data to support this claim. Already in the first report of fibrils in brains from prion infected mice, there was a consistent presence of fibrils also in clinically silent animals^28^. Prions hence likely exist in both replicating and neurotoxic conformations^53^ and this can influence how the host reacts upon prion infection. This also suggests that prions in a silent carrier can be transmitted to a disease susceptible host^54^. While it is beyond the scope of this study one can speculate on the pre-requisites for neurotoxicity in relation to amyloidogenicity given that the latter could be omnipresent in prion susceptible and resistant species. The predicted amyloidogenic sequence 171-176 includes part of the previously well studied “rigid loop” within native PrP which is sensitive to substitutions and affects PrP misfolding^18^,^55^,^56^. The amyloidogenic 171-176 amino acid stretch is present in four of five PrPs. N is substituted for S in position 174 of PoPrP and this stretch is not predicted as amyloidogenic by WALTZ (Fig. 6b) which suggestively could affect downstream neurotoxicity in pigs. A survey of prnp genes from different carnivores implied that prion disease resistant canines harbor amino acids DRK in positions 159, 177, and 185 while the prion susceptible felines as well as mustelines (e.g. mink) predominantly express NHR (feline) or NHK (musteline) in the corresponding positions^57^. The BoPrP, HuPrP, MoPrP, PoPrP and HaPrP sequences (all prion susceptible) are identical to the prion susceptible musteline at these positions indicating susceptibility to prion disease. These positions are flanking the stretches indicative of amyloidogenicity according to WALTZ (Fig. 6b) and are hypothetically important for disease and hence as facilitating toxic signaling from PrP interacting with misfolded PrP. If PrP misfolding is necessary but not sufficient to cause prion disease our data herein showing generic amyloidogenicity of mammalian PrPs albeit with different efficiencies suggests that species considered being prion resistant may potentially harbor replicating prions while lacking other downstream effects in relation to disease. This possibility merits further studies.

## Methods

### Protein expression and purification

Plasmids (pRSET vector) containing the genes of interest were kindly provided by Dr S Hornemann^12^. HaPrP was generated by site directed mutagenesis according to manufacturer’s recommendations (QuikChangeII, Agilent) using MoPrP as template. The gene constructs were cloned with a hexaHis tag to facilitate Ni^2+^affinity chromatography. Proteins were expressed and purified as previously reported^25^. In brief, the plasmid was transformed into BL21/DE3 E.Coli cells, cultured in TB medium to OD ~ 3 where after protein synthesis was induced by addition of 1 mM isopropyl-thiogalactose and protein production was left to proceed at 37 °C over-night. Cells were harvested by centrifugation and the pellet was stored at −80 °C until needed. Cells were lysed by sonication in buffer containing 6 M guanidine hydrochloride (GuHCl), cell debris were removed by centrifugation and the supernatant was applied to Ni-NTA agarose beads (Qiagen). The beads were washed, the protein was refolded and the disulfide bond was oxidized on the column as described in Zahn *et al.*, 1997^58^ prior to elution with 500 mM imidazole at pH 5.8. Subsequent size exclusion chromatography using a Hiload 16/60 superdex 75 prep grade column and buffer F (50 mM phosphate (pH 7.4), 100 mM NaCl, 50 mM KCl) as running buffer was performed to ensure purification of native monomeric protein. Proteins were stored at 4 °C for a maximum of 7 days prior to use. Protein concentration was determined by measuring absorbance at 280 nm using the theoretical extinction coefficients; HuPrP 57995 M^−1^cm^−1^ , BoPrP 63495 M^−1^cm^−1^, PoPrP 59485 M^−1^cm^−1^, FePrP 59485 M^−1^cm^−1^, CaPrP 59485 M^−1^cm^−1^, MoPrP 63495 M^−1^cm^−1^, and HaPrP 62005 M^−1^cm^−1^ as calculated from ExPASy ProtParam tool at the Swiss Institute of Bioinformatics website http://web.expasy.org/protparam/59.

### Circular dichroism

Samples were dialyzed 3x against buffer F supplemented with 20 mM EDTA and 2x against buffer F using Slide-a-lyzer 3500 MWCO (Pierce) prior to CD measurements to remove residual imidazole. A Chirascan CD spectrometer (Applied photophysics) and 1 mm cuvette was used for all measurements. Far UV spectra (between 200 and 250 nm) were measured at 4 °C to assess the native protein structure. Thermal denaturation was monitored by recording the molar ellipticity at 222 nm while the sample was heated from 4 °C to 94 °C at 1 °/min. The respective T_m_ was determined by calculating the maximum of the derivative in the denaturation transition according to John *et al.*^60^

### Fibrillation assay

The proteins were diluted in buffer F to a final PrP concentration of 5 μM and supplemented with ThT to a final concentration of 2 μM. 100 μl samples were aliqouted in black non-treated half-area plates with transparent bottom (Corning costar). 1% seed (i.e. 1 μl to 100 μl sample) was added as required for each experiment. The plates were sealed with transparent sealing tape before being placed in a Tecan SafireII plate reader. The proteins were subjected to the Native Condition Conversion Assay (NCCA)^25^. Herein the plate was continuously, linearly shaken at highest speed (1584 shakes/min with 1 mm amplitude) and halted every 15 minutes to measure fluorescence intensity at 480 nm from the bottom of the plate (excitation at 440 nm) providing an average value from 10 scans per well for each time point. Similar protocols devoid of denaturing agents have been used for fibrillation of HuPrP^26^ and MoPrP^61^. Monitoring was continued for 24 hours or, if conversion had not occurred after 24 hours, for 48 hours. Fibrils to be used as seeds in seeding experiments were produced according to the same procedure but without addition of ThT. Seeds were prepared from 6 pooled fibrillation reactions. Amyloid content of the seed solutions was verified by *ex situ* ThT staining at the end point both for PrPs and the other amyloid proteins. Amyloid fibrils from chicken Lysozyme was produced by incubation of 200 μM protein for 1 week in 25 mM HCl at 65 °C, for bovine insulin by incubation of 320 μM protein in 25 mM HCl at 65 °C for 24 h, TTR fibrils were made by incubation of 350 μM protein for 2 weeks in 50 mM acetic acid pH 3.0, 100 mM NaCl at 4 °C. Fibrils of Aβ 1-40 and Aβ 1-42 was made by incubation of 10 μM protein in PBS, pH 7.4 at 37 °C for 48 h. All fibrils were dialyzed to buffer F prior to use seeds to mimic PrP seeding. Control proteins (Bovine serum albumin (BSA), Bovine carbonic anhydrase II (BCAII), Bovine immunoglobulin G (BoIgG), Horse myoglobin and Chicken egg white lysozyme, all from Sigma) were dissolved in PBS to a final concentration of 6 μM and subjected to the same fibrillation conditions as described above for PrPs.

### Congo red staining and microscopy

Congo red stock solution was added to mature fibrils in solution at a molar ratio of PrP:dye 10:1. Stained fibrils were left to self-sediment over-night at 4 °C. 4 μl from the bottom of the pelleted samples were transferred to superfrost glass slides (Thermo Fisher, Walldorf, Germany) and allowed to dry. The dried droplets were covered with fluorescence mounting medium (Dako, Glosrup, Denmark) and cover glass edges were sealed with transparent nail polish. Congo red stained samples were analyzed using a Nikon light microscope equipped with polarizers for both incoming light and in front of the eyepiece/camera. Staining with hFTAA was performed in an analogous manner as for Congo red using 300 nM hFTAA. The stained PrP aggregates were imaged by hyperspectral fluorescence using a Leica DM6000 B fluorescence microscope (Leica, Wetzlar, Germany) equipped with a SpectraCube module (Applied Spectral Imaging, Migdal Ha-Emek, Israel) as described in^25^.

### Transmission electron microscopy

For electron microscopy, samples were applied to carbon coated copper mesh grids (Ted Pella, inc.). In detail 5 μl aliquots PrP samples subjected to fibrillation conditions in the absence or presence of seeds for 24 h (HuPrP, FePrP, CaPrP, PoPrP, MoPrP, HaPrP) or 48 h (BoPrP) was applied for 2 min. The grid was blotted dry and rinsed with 5 μl of dH_2_O, followed by counterstaining with 5 μl of with 2% (w/v) uranyl acetate dissolved in dH_2_O for 30 s. Micrographs were collected using a Jeol 1230 transmission electron microscope operating at 100 kV equipped with a CCD camera.

### Aggregation prediction algorithms

The AGGRESCAN algorithm to predict aggregation prone linear segments was run from the website http://bioinf.uab.es/aggrescan/46. WALTZ predictions of the PrP sequences to assess stretches of linear amyloid forming sequences was applied using the WALTZ prediction tool using the high specificity threshold for scoring and pH 7.0. The WALTZ algorithm was run from the website http://waltz.switchlab.org/47.

## Additional Information

**How to cite this article**: Nyström, S. and Hammarström, P. Generic amyloidogenicity of mammalian prion proteins from species susceptible and resistant to prions. *Sci. Rep.* **5**, 10101; doi: 10.1038/srep10101 (2015).

## Supplementary Material

Supplementary Information

## Figures and Tables

**Table 1:** Amyloid-like features of aggregated PrPs.

**Sequence**	**TEM**	**CR**	**hFTAA**	**ThT kinetics**
BoPrP	-	+	+	+
HuPrP	+	+	+	+
FePrP	+	+	+	+
MoPrP	+	+	+	+
PoPrP	+	+	+	+
HaPrP	+	+	+	+
CaPrP	+	+	+	+

TEM: Fibrillar structure detected by transmission electron microscopy (Fig. 1)

CR: Displays red green birefringence when stained with Congo red (Fig. 2a)

hFTAA: hFTAA positive aggregates displaying amyloid spectrum when monitored by fluorescence microscope (Fig. 2b)

ThT kinetics: Sigmoidal kinetic profile of ThT fluorescence (Fig. 4b)
