# Supplementary material for: Generic amyloidogenicity of mammalian prion proteins from species susceptible and resistant to prions
Source: Sci Rep. 2015 May 11;5:10101. doi: 10.1038/srep10101 (PMC4650755; doi:10.1038/srep10101)
Supplement: Supplementary Information [file srep10101-s1.pdf]

Supporting information

Generic amyloidogenicity of mammalian prion proteins from species susceptible and resistant to prions

*Sofie Nyström and Per Hammarström**

IFM-Department of Chemistry, Linköping University, SE-581 83 Linköping, Sweden

*corresponding author e-mail: perha@ifm.liu.se

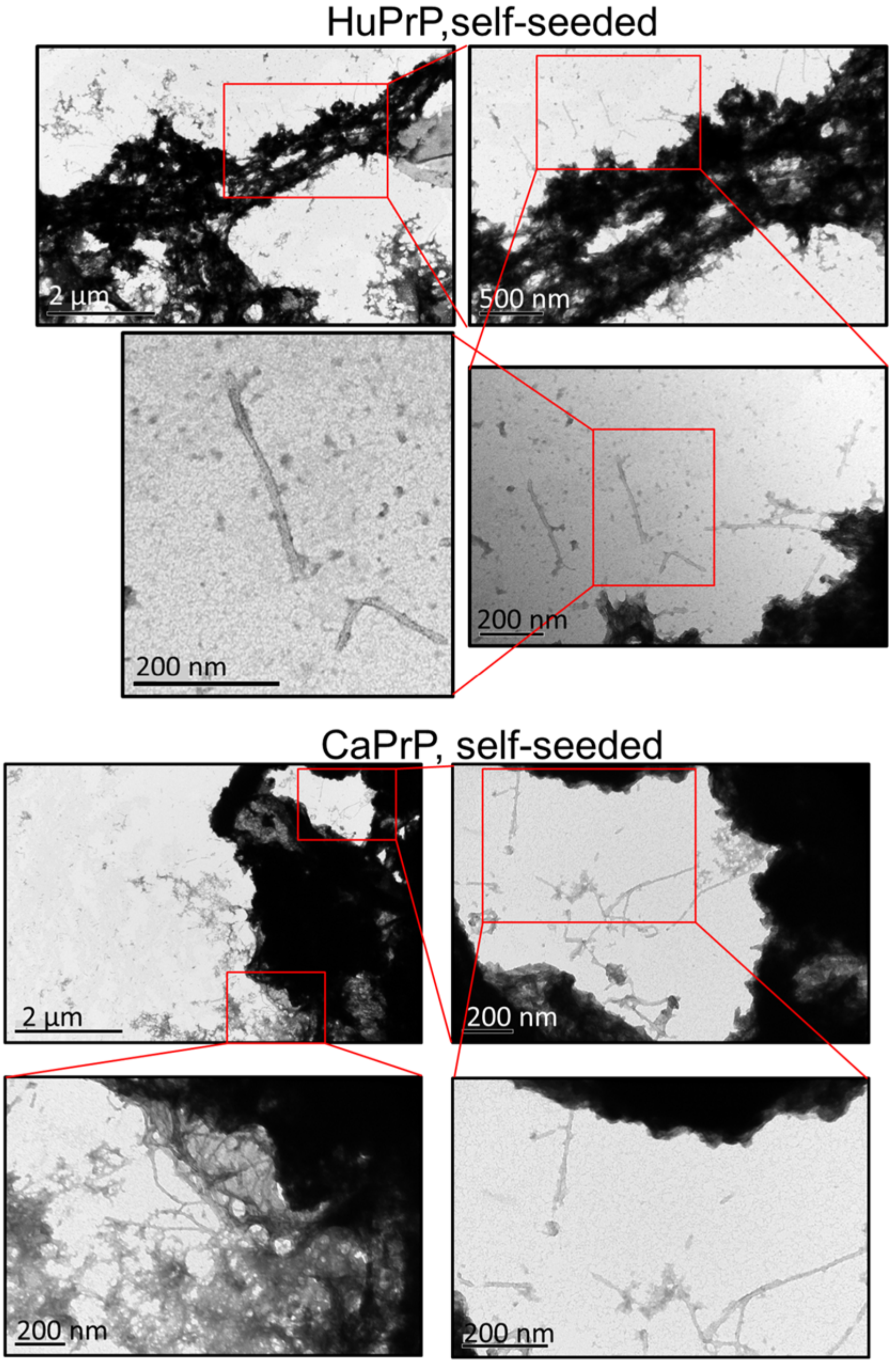

FIGURE S1: **Examples of ultrastructure of seeded mammalian PrP aggregates by transmission electron microscopy.** HuPrP and CaPrP displayed common features upon seeding showing fibrils protruding from heavily clustered aggregates.

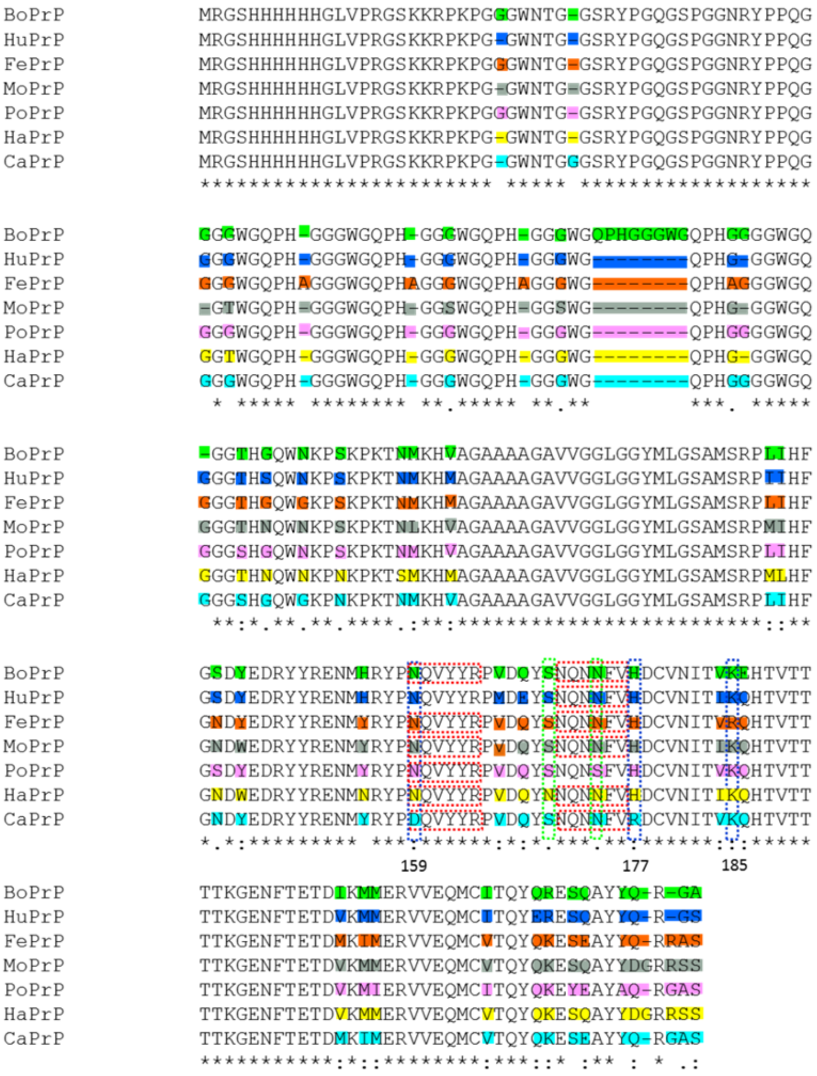

FIGURE S2 **Sequence alignments of mamPrPs**. Stars indicate identical residues. Stars indicate identical residues colon denotes conserved residues and points mark non-conserved mutations. Mismatched residues are marked in color. The red boxes highlight amyloidogenic stretches according to the WALTZ prediction tool [47] and the blue boxes highlight amino acids modifying disease susceptibility according to [57].
